# The spells of iatrogeny

**DOI:** 10.1007/s12471-023-01761-8

**Published:** 2023-02-02

**Authors:** A. F. Cardoso, G. Dias, B. Faria, F. Almeida, A. Lourenço

**Affiliations:** Department of Cardiology, Hospital Senhora da Oliveira—Guimarães, Guimarães, Portugal

A 50-year-old female presented to the emergency department with complaints of tiredness, atypical chest pain, nausea and headache for the last 3 days. She had a 2-year history of arterial hypertension. On admission, her blood pressure was 120/70 mm Hg, her heart rate was 100 bpm and she was apyretic. Physical examination was unremarkable. The electrocardiogram (ECG) showed a sinus rhythm with poor R wave progression and biphasic T waves in the precordial leads. Blood tests revealed elevated levels of high-sensitivity troponin I (745 ng/l; reference value < 45) and N‑terminal-prohormone brain natriuretic peptide (5000 pg/ml; reference value < 125). Transthoracic echocardiography showed moderate left ventricular dysfunction with akinesis of mid and apical segments.

She was admitted with the diagnosis of probable Takotsubo cardiomyopathy. ECG evolved with deep inverted T waves on precordial leads. She was started on beta-blocker therapy (carvedilol 6.25 mg), and intravenous metoclopramide was administered for nausea control. Soon thereafter, we observed significant clinical worsening. The patient evolved with pallor, diaphoresis, worsening headache and palpitations. ECG and invasive blood pressure monitoring are represented in Fig. 1a. Echocardiography showed superimposed left ventricular alterations. In subcostal view, a heterogeneous mass with well-defined borders, measuring approximately 48 × 61 mm, was visible under the liver (Fig. 1b and see Video 1 in Electronic Supplementary Material).

**Fig. 1:**
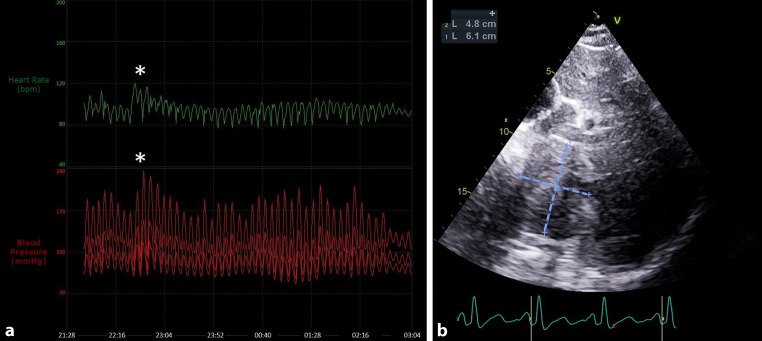
**a** Heart rate and invasive blood pressure monitoring documenting paroxysms of tachycardia and arterial hypertension over a period of approximately 5 h (*asterisk* denotes highest recorded value of 120 bpm for heart rate and 240/120 mm Hg for blood pressure). **b** Echocardiographic subcostal view showing a heterogeneous mass with well-defined borders (size: ~48 × 61 mm) under the liver

What is the most likely diagnosis?

## Answer

You will find the answer elsewhere in this issue.

## Supplementary Information

**Video 1 **Transthoracic echocardiogram, apical 4‑chamber view

